# Aberrant overexpression of ADAR1 promotes gastric cancer progression by activating mTOR/p70S6K signaling

**DOI:** 10.18632/oncotarget.13354

**Published:** 2016-11-15

**Authors:** Ning Dou, Shijun Yu, Xiaojuan Ye, Dong Yang, Yandong Li, Yong Gao

**Affiliations:** ^1^ Department of Oncology, Shanghai East Hospital, Tongji University School of Medicine, Shanghai 200120, China; ^2^ Research Center for Translational Medicine, Shanghai East Hospital, Tongji University School of Medicine, Shanghai 200120, China

**Keywords:** ADAR1, gastric cancer, tumorigenecity, metastasis, mTOR

## Abstract

ADAR1, one of adenosine deaminases acting on RNA, modulates RNA transcripts through converting adenosine (A) to inosine (I) by deamination. Emerging evidence has implicated that ADAR1 plays an important role in a few of human cancers, however, its expression and physiological significance in gastric cancer remain undefined. In the present study, we demonstrated that ADAR1 was frequently overexpressed in gastric cancer samples by quantitative real-time PCR analysis. In a gastric cancer tissue microarray, ADAR1 staining was closely correlated with tumor stage (*P* < 0.001) and N classification (*P* < 0.001). Functional analysis indicated that ADAR1 overexpression promoted cell proliferation and migration *in vitro*, whereas ADAR1 knockdown resulted in an opposite phenotypes. Furthermore, ADAR1 knockdown also inhibited tumorigenicity and lung metastasis potential of gastric cancer cells in nude mice models. Mechanistically, ADAR1 expression had a significant effect on phosphorylation level of mTOR, p70S kinase, and S6 ribosomal protein, implying its involvement in the regulation of mTOR signaling pathway. We conclude that ADAR1 contributes to gastric cancer development and progression via activating mTOR/p70S6K/S6 ribosomal protein signaling axis. Our findings suggest that ADAR1 may be a valuable biomarker for GC diagnosis and prognosis and may represent a new novel therapeutic opportunities.

## INTRODUCTION

Although there are many hypotheses about the formation of malignancies, the exact pathogenesis of tumor still remains unknown. Currently, the most convincible mechanisms are changes on DNA level, including DNA mutation, gene amplification or loss, DNA methylation and covalent histone modification [1]. Until more recently, with the application of NGS-based RNA-Seq, evidences have revealed a global alterations of RNA information in entire transcriptomes [2]. The RNA alterations especially derived from RNA editing is pervasive, with more than 85% of RNAs likely to be edited in the coding or non-coding regions [3–5]. Accumulating reports have shown that dysregulation of RNA editing may contribute to various diseases including cancer [6, 7].

RNA editing is a post-transcriptional event that generates RNA and protein diversity by insertion, deletion, and base substitution of nucleotides within the edited RNA molecule. The most prevalent type of RNA editing in higher eukaryotes is adenosine (A) to inosine (I) editing which can be highly selective [8–11] or occur at multiple sites [12, 13]. Importantly, A-to-I editing could alter several biological processes because I is recognized as G instead of A. These processes include mRNA translation, splicing site recognition and RNA structure-dependent activities. In general, A-to-I editing is catalyzed by the adenosine deaminases acting on RNA (ADARs) class of enzymes and it usually occurs in regions of double-stranded RNA (dsRNA). Mammalian ADAR family comprises three members: ADAR1, 2 and 3. All ADARs have a highly conserved C-terminal catalytic deaminase domain and contain several N-terminal dsRNA-binding motifs. ADAR1 and ADAR2 are found in many tissues in the body while ADAR3 is exclusively detected in the central nervous system [14, 15]. Only ADAR1 and ADAR2 were shown to be active for RNA editing. There are two isoforms of ADAR1, an IFN-inducible ∼150kDa form (p150) found in both the cytoplasm and nucleus and a constitutively expressed ∼110kDa form (p110), found predominantly if not exclusively in the nucleus [16, 17].

Scientists have done some researches to investigate the role of ADAR1 in tumor and tried to figure out how ADAR1 is working in malignance. Upregulation of ADAR1 was discovered in a few of malignant tissues, such as hepatocellular carcinoma [18], breast cancer [19] and esophageal squamous cell carcinoma [20]. One report has demonstrated that ADAR1 undergoes gene amplification associated with overexpression of the transcript and protein in lung cancer, which enhances human lung tumorigenesis [21]. By contrast, downregulation of ADAR1 was found in brain cancers [22] and metastatic melanomas [23, 24]. In hepatocellular carcinoma, ADAR1 could increase editing level in the coding sequence of AZIN1 [18], thereby stabilizing this protein and promoting cell proliferation. In addition to RNA editing in coding sequences, other reports have also indicated that several tumor-related microRNAs were edited by ADAR1, leading to miRNA biogenesis disorder or change of their targeted mRNA [25–28]. Given the evidence provided in previous reports, ADAR1 might be a new diagnostic, therapeutic indicator and prognostic prediction in tumor, but its exact roles and underlying mechanism in tumorigenesis are still to be classified.

Gastric cancer (GC) is one of the major malignancies and the third cause of cancer-related death in the world [29]. It often responds poorly to current therapeutic approaches and is frequently associated with a poor prognosis. This study was to elucidate the expression level of ADAR1 in gastric carcinoma tissues, the clinical relevance, and the effect of ADAR1 on GC cell growth and metastasis *in vitro* and *in vivo*. We finally found ADAR1 is frequently upregulated in GC and its expression is closely associated with GC progression of patients. Moreover, we provide evidence that ADAR1 promotes GC cell growth and migration by a possible mechanism involving mTOR/p70S6K/S6 ribosomal protein signaling pathway.

## RESULTS

### ADAR1 is frequently upregulated in GC

**Figure 1:**
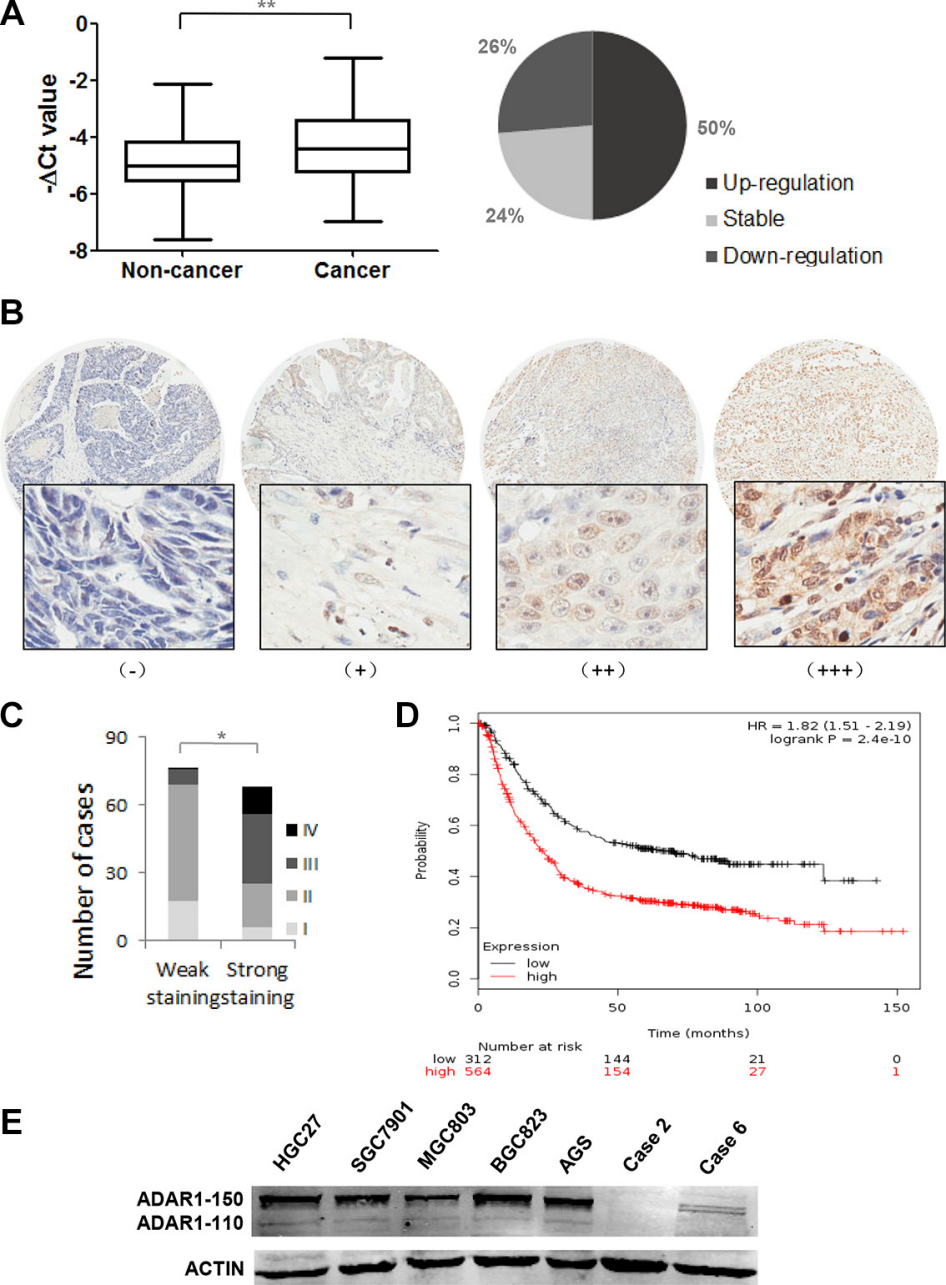
Expression pattern of ADAR1 in gastric cancer tissues and cell lines (**A**) The mRNA expression of ADAR1 was measured in 38 paired gastric cancer samples and adjacent, non-cancerous stomach tissues by quantitative real-time-PCR. Data is shown as –ΔCt, where β-actin was used as internal control. *P value* was calculated by Student's *t* test, **P < 0.01. (**B**) The protein expression of ADAR1 was performed with immunohistochemical staining on a gastric cancer tissue microarray with anti-ADAR1 antibody. (−), representative sections of negative staining; (+), slight positive; (++), moderate positive; (+++), strong positive. Magnification: × 40 (upper) and × 200 (bottom). (**C**) The positive ratio of ADAR1 staining in TNM stage I, II, III and IV. Weak staining includes (−) and (+); strong staining includes (++) and (+++). *P < 0.01. (**D**) Kaplan-Meier overall survival plot comparing patients demonstrating high ADAR1 expression (black line; *n* = 564) and low ADAR1 expression in tumors (red line; *n* = 115; *P* < 0.01, Log-rank test). (**E**) The expression of ADAR1 was evaluated by western blot in five gastric cancer cell lines and two random adjacent non-cancerous stomach tissues.

ADAR1 expression was initially evaluated in 38 paired human gastric cancer specimens by quantitative real-time PCR. The mRNA level of ADAR1 was significantly upregulated in GC specimens, as compared to that of corresponding noncancerous stomach tissues. 19/38 (50%) of the GC specimens were observed at more than 1.5-fold increase (Figure 1A). Furthermore, a tissue microarray containing 143 patients’ samples with available clinical information was examined by immunohistochemical staining with a specific antibody against ADAR1. Among them, ADAR1 protein exhibited weak staining in early stage (stage I and stage II) and strong staining in late stage (stage III and stage VI) (Figure 1B, 1C and Table 1, p < 0.001). There was also a significant correlation between ADAR1 staining and N classification (Table 1, p < 0.001). Importantly, by analyzing Kaplan-Meier Plotter datasets, high ADAR1 expression was found to be significantly associated with poor outcomes of gastric cancer patients, overall survival of which was markedly decreased (Figure 1D). These clinicopathological data suggested a strong association of ADAR1 expression with GC progression. To validate these findings, we used western blot analysis to examine ADAR1 expression in gastric cancer cell lines. The results showed that ADAR1 was highly expressed in AGS, BGC823 and HGC27 cells relative to the MGC803 cell line and two cases of adjacent non-cancerous tissues (Figure 1E), supporting the notion that ADAR1 was frequently overexpressed in GC.

**Table 1:** The correlation of ADAR1 expression with clinicopathological features of gastric cancer

	Number of cases	weak staining	strong staining	*P* value
Age (years)				
> 60	90	44	46	0.121
≤ 60	53	33	20	
Gender				
Male	97	51	46	0.659
Female	46	26	20	
Differentiation				
Well-differentiated	14	8	6	0.920
Moderately differentiated	47	26	21	
Poorly differentiated	82	43	39	
T classification				
T1,T2	26	21	5	0.002
T3,T4	117	56	61	
N classification				
N0	96	65	31	< 0.001
N1-3	47	12	35	
M classification				
M0	142	77	65	0.278
M1	1	0	1	
Stage				
I, II	95	69	26	< 0.001
III, IV	48	8	40	

### ADAR1 promotes GC cell growth and colony formation *in vitro*

**Figure 2:**
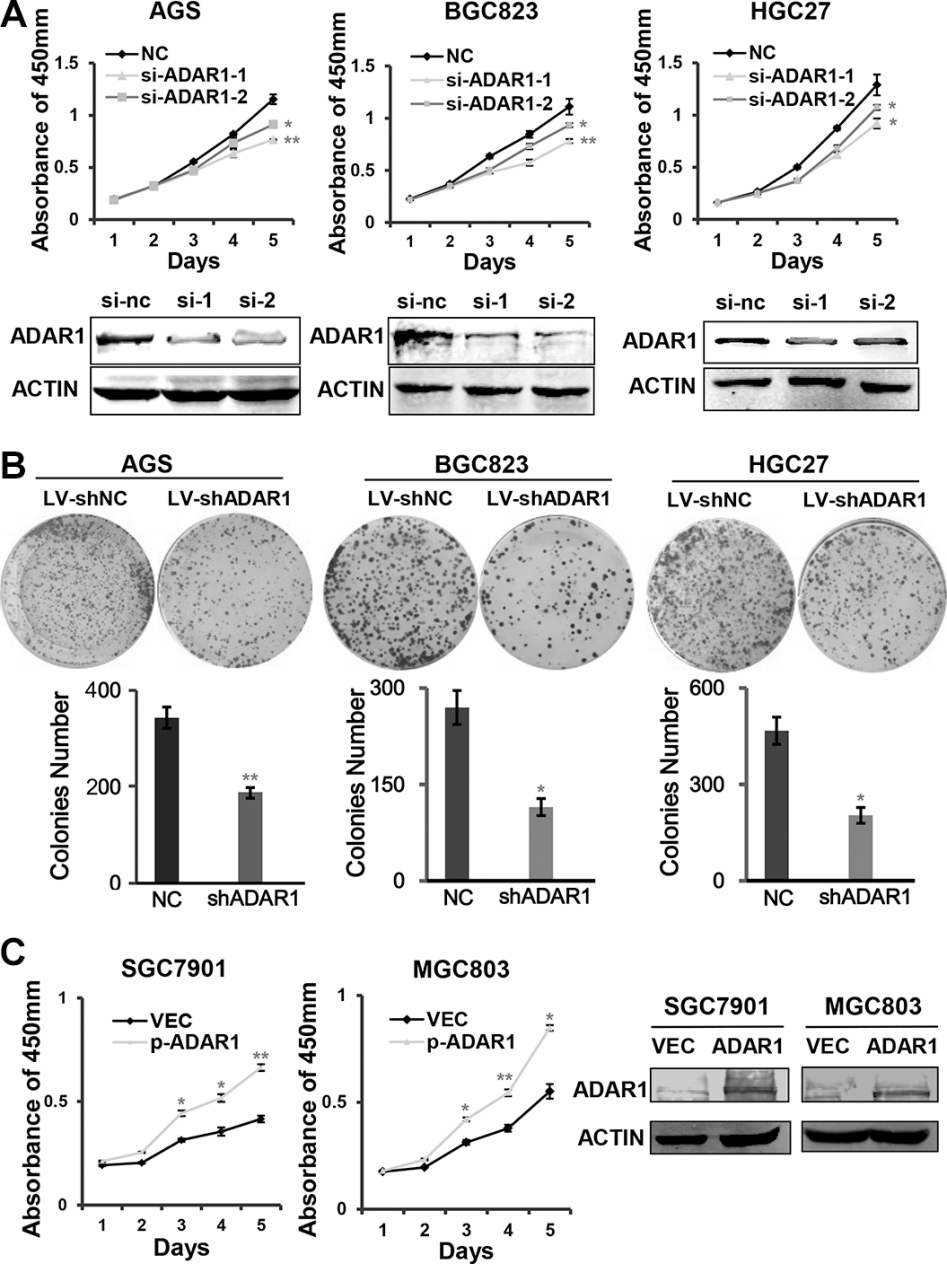
ADAR1 enhanced GC cell growth and colony formation (**A**) The effects of ADAR1 knockdown on cell proliferation in AGS, BGC823 and HGC27 cells. The knockdown efficiency of ADAR1 was determined by western blotting. (**B**) The effects of ADAR1 knockdown on colony formation capacity of AGS, BGC823 and HGC27 cells. The same amounts of related cells were plated into a 6-well plate. Cell colonies were stained and counted after two weeks. (**C**) Cell growth was examined by CCK8 method in ADAR1 overexpressed SGC7901 and MGC803 cells. Western blot analysis was used to detect ADAR1 expression in the two cell lines. All the above experiments were repeated at least three times. The data represents mean ± SD of three independent experiments. *P < 0.05, **P < 0.01.

Since ADAR1 is significantly upregulated in GC, we next determined whether ADAR1 functions as an oncogene. CCK-8 method was employed to assess cell growth ability after silencing ADAR1. The small interference RNAs were chemically synthesized and transiently transfected into AGS, BGC823 and HGC27 cells, respectively. Western blot results indicated that ADAR1 was successfully knocked down in these cells (Figure 2A). Cell growth curves showed those cells transfected with siADAR1-1 and siADAR1-2 displayed a significant growth inhibition compared with those cells transfected with siNC in the three cell lines examined (Figure 2A). Furthermore, we also stably infected the three gastric cancer cell lines with interference lentivirus LV-shADAR1 or with control LV-shNC. Colony formation assays indicated that knockdown of ADAR1 resulted in fewer and smaller colonies in LV-shADAR1 group than those in LV-shNC group (Figure 2B). On the other hand, ADAR1 overexpression significantly promoted GC cell growth in both MGC803 and SGC7901 cells (Figure 2C). These collective observations implicated that ADAR1 facilitates GC cell growth *in vitro*.

### Silencing of ADAR1 induces cell death and growth inhibition of GC cells

**Figure 3:**
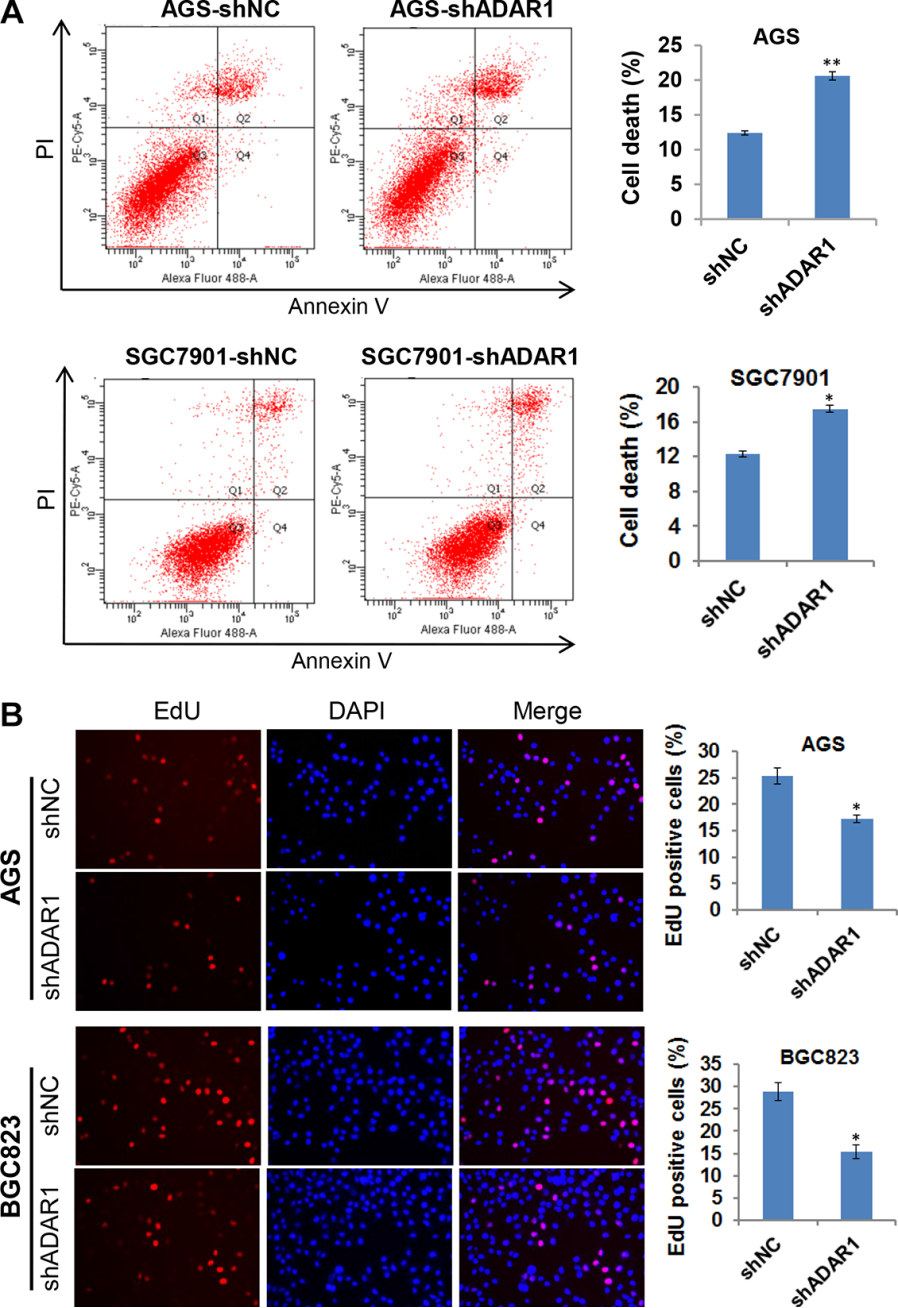
Knockdown of ADAR1 induced cell death and slowed down cell growth (**A**) Cell apoptosis was examined by Annexin-V/PI staining and flow cytometry analysis in two groups of cells stably silencing ADAR1 (AGS/shNC and AGS/shADAR1, SGC7901/shNC and SGC7901/shADAR1). The proportion of cell death was determined by PI-positive staining. Error bars represent mean ± SD for three independent experiments. *p < 0.05, **p < 0.01. (**B**) EdU incorporation assay was used to measure cell proliferation in AGS/LV-shADAR1, BGC823/LV-shADAR1 cells and their corresponding control cells. Data represented as mean ± SD deviation of three independent experiments. *p < 0.05.

During the cell culture, we can see more floating cells in the cell culture supernatant of ADAR1 knockdown cells. Thus, flow cytometric analysis was performed to determine whether ADAR1 knockdown affects cell apoptosis. Interestingly, no obvious difference was found between ADAR1 knockdown cells and controls cells in the early apoptosis stage (PI-negative and Annexin V-positive), but the number of dead cells (PI-positive) significantly increased in the AGS-LV-shADAR1 and SGC7901-LV-shADAR1 cells compared with respective controls (Figure 3A), indicating that ADAR1 konckdown strongly induced GC cell death. Moreover, EdU incorporation assay was also performed in AGS-LV-shADAR1 and BGC823-LV-shADAR1 cells. As shown in Figure 3B, knockdown of ADAR1 in AGS and BGC823 cells decreased in the percentage of EdU positive cells, suggesting that ADAR1 knockdown suppressed cell proliferation. These findings suggested that ADAR1 is required for cell viability and proliferation in GC.

### ADAR1 enhances migration of GC cells *in vitro*

**Figure 4:**
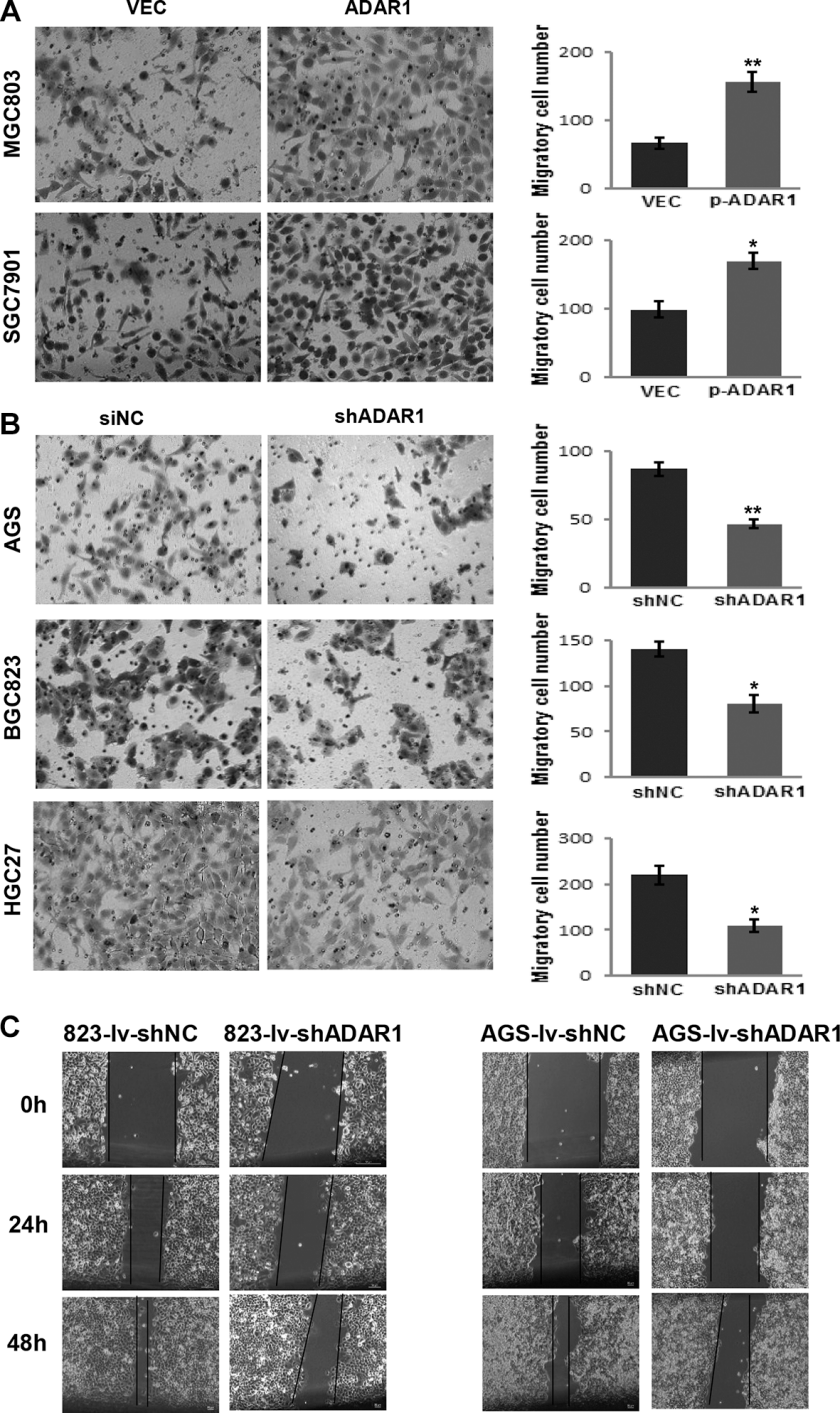
ADAR1 promotes GC cell motility (**A**) The migration abilities of MGC803 and SGC7901 cells transfected with ADAR1 overexpression plasmid or empty vector were evaluated by transwell chamber assays, respectively. All experiments were repeated at least three times. Representative fields of migrated cells are shown. Cell numbers were counted in five randomly selected microscopic fields and the data are shown as the mean ± SD. *P < 0.05, **p < 0.01. (**B**) Migration of AGS, MGC823 and HGC27 cells was evaluated by transwell assay after stably infected with LV-shADAR1, where LV-shNC was used as a control. Cell numbers were counted in five randomly selected microscopic fields and the data is shown as mean ± SD of three independent experiments. *P < 0.05, **p < 0.01. (**C**) Wound healing experiment was used to analyze the motility ability of BGC823 and AGS cells in which ADAR1 was silenced.

To further characterize the effect of ADAR1 in GC, we tested cell migration by using transwell assay and wound healing assay. As shown in Figure 4A, compared with cells that were transfected with empty vector, ADAR1 overexpression significantly promoted cell migration in both MGC803 and SGC7901 cells. Concordantly, ADAR1 knockdown strongly reduced the migrated cell number in another three cell lines AGS, BGC823 and HGC27 when compared with the controls (Figure 4B). Wound healing assay subsequently confirmed that ADAR1 knockdown could restrain the motility of BGC823 and AGS cells (Figure 4C). These results suggested that ADAR1 plays a role in promoting cell migration in GC.

### Silencing of ADAR1 attenuates the tumorigenicity and metastasis of GC cells *in vivo*

**Figure 5:**
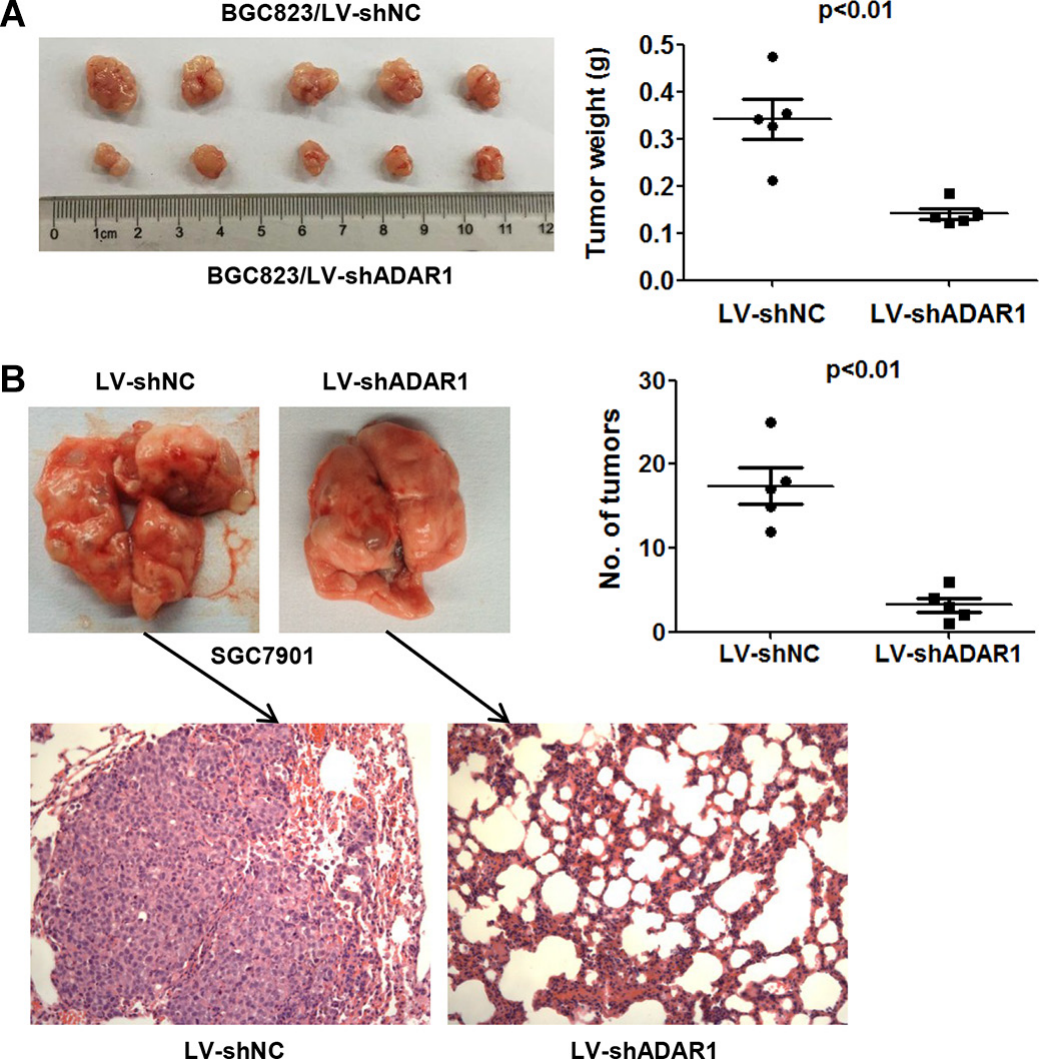
ADAR1 knockdown suppressed the tumorigenicity and metastasis of GC cell in nude mice (**A**) The impact of ADAR1 knockdown on tumorigenicity of BGC823 cells in nude mice. Animals were sacrificed and tumor tissues were collected and photographed, tumor weight was measured at the end of the experiment. Values are expressed as mean ± SEM (*n* = 5). (**B**) Lung metastasis potential assay for ADAR1 knockdown was examined in SGC7901 cells by tain vein injection. The tumor nodules were observed in lung surface after 40 days receiving 1.5 × 10^6^ SGC7901-shNC or SGC7901-shADAR1 cells. The mean number of tumor nodules from each group (*n* = 5) and the hematoxylin and eosin (HE) stained lung sections are shown. Magnification: ×200.

Based on our findings *in vitro*, we performed tumor xenograft assays to examine whether ADAR1 knockdown could decrease the tumorigenicity and metastasis *in vivo*. The stable cells BGC823/LV-shADAR1 and control BGC823/LV-shNC were subcutaneously injected into four-week old male nude mice respectively (*n* = 5). As expected, the tumors grew more slowly and the average tumor weight was lower in the BGC823/LV-shADAR1 group than those in the BGC823/LV-shNC group (Figure 5A). For metastasis assay *in vivo*, SGC7901 cells were employed and injected directly into tail veins of nude mice (*n* = 5). The final results showed that silencing of ADAR1 markedly decreased the nodule number on the lung surface from mice receiving SGC7901/LV-shADAR1 cells than that of the control cell SGC7901/LV-shNC. Histological analysis confirmed the presence of metastatic tumors in the lungs of these mice (Figure 5B). Taken together, these data suggested that ADAR1 is involved in the positive regulation of tumorigenicity and metastasis of GC cells *in vivo*.

### ADAR1 affects mTOR signaling pathway activities

**Figure 6:**
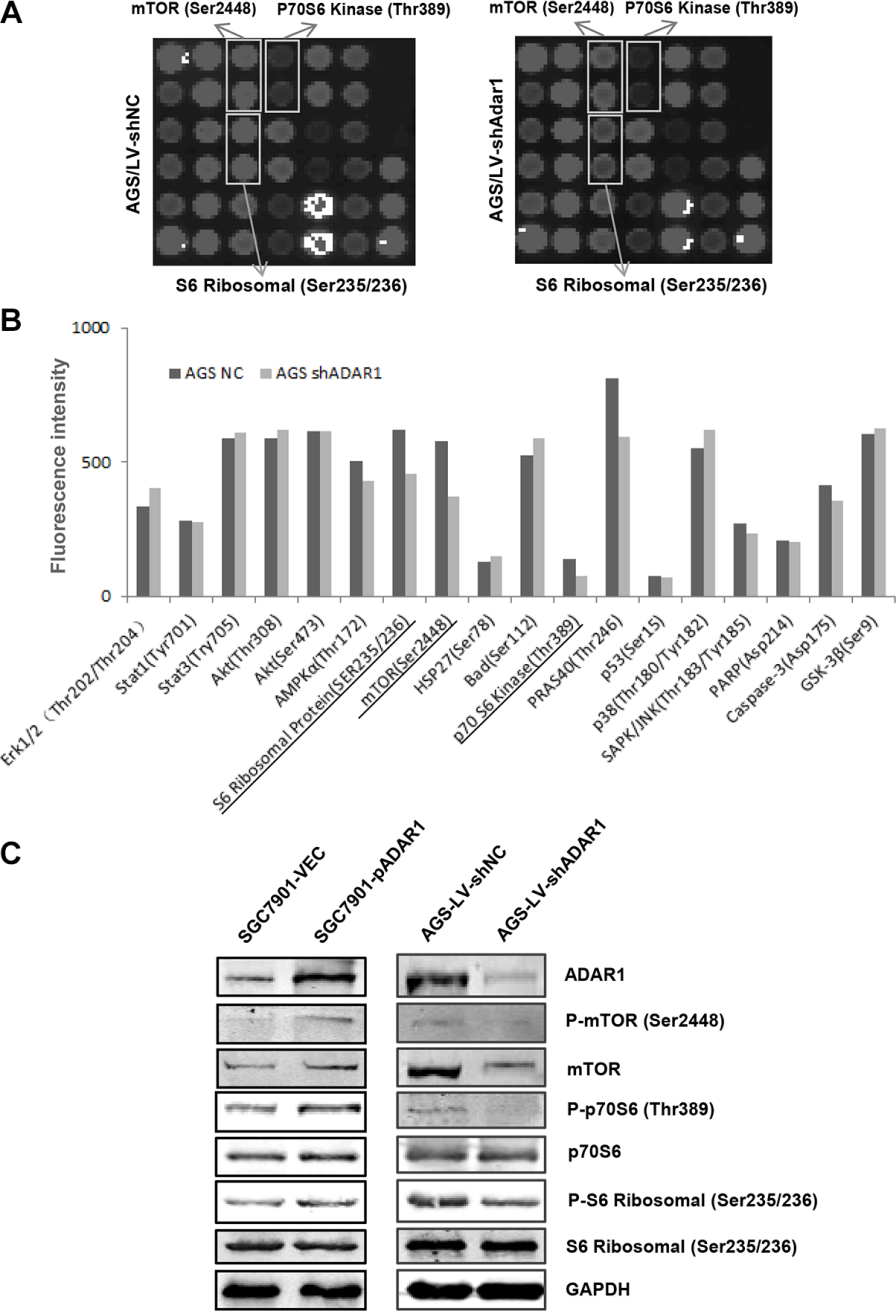
Effects of ADAR1 on the phosphorylation level of mTOR signaling in GC cells (**A**) Intracellular signaling array was performed in AGS cells before and after silencing ADAR1. The significantly changed protein dots were marked with rectangles. (**B**) Data quantified by Image Studio Version 3.1 for each protein dots of intracellular signaling array used above. (**C**) Western blot analysis was used to confirm the phosphorylation level change of mTOR, p70S6 kinase and S6 ribosomal protein in SGC7901 cells (for ADAR1 overexpression) and AGS cells (for ADAR1 knockdown).

To figure out the molecular mechanisms of how ADAR1 promotes GC cell growth and migration, we used a PathScan^®^ intracellular signaling array kit to compare cell signaling in AGS cells before and after ADAR1 knockdown. As shown in Figure 6A and 6B, the phosphorylation of mTOR (Ser2448), phosphorylation of p70S6 kinase (Thr389) and phosphorylation of S6 ribosomal protein (Ser235/236) were downregulated in AGS/LV-shADAR1 cells compared with the control cells. This result was further confirmed by Western Blot in AGS/LV-shADAR1 and AGS/LV-shNC cells (Figure 6C). Meanwhile, when ADAR1 was overexpressed in SGC7901 cells, the phosphorylation levels of mTOR, p70S6 kinase and S6 ribosomal protein were significantly increased in accordance with the reduction induced by ADAR1 knockdown (Figure 6C). As known, mTOR, p70S6 kninase and S6 ribosomal protein constitute an important signaling axis involved in the regulation of tumor growth and metastasis. Our results provided a possible mechanism that ADAR1 enhances GC cell proliferation and migration through mTOR/p70S6 Kinase/ S6 ribosomal protein pathway.

**Figure 7:**
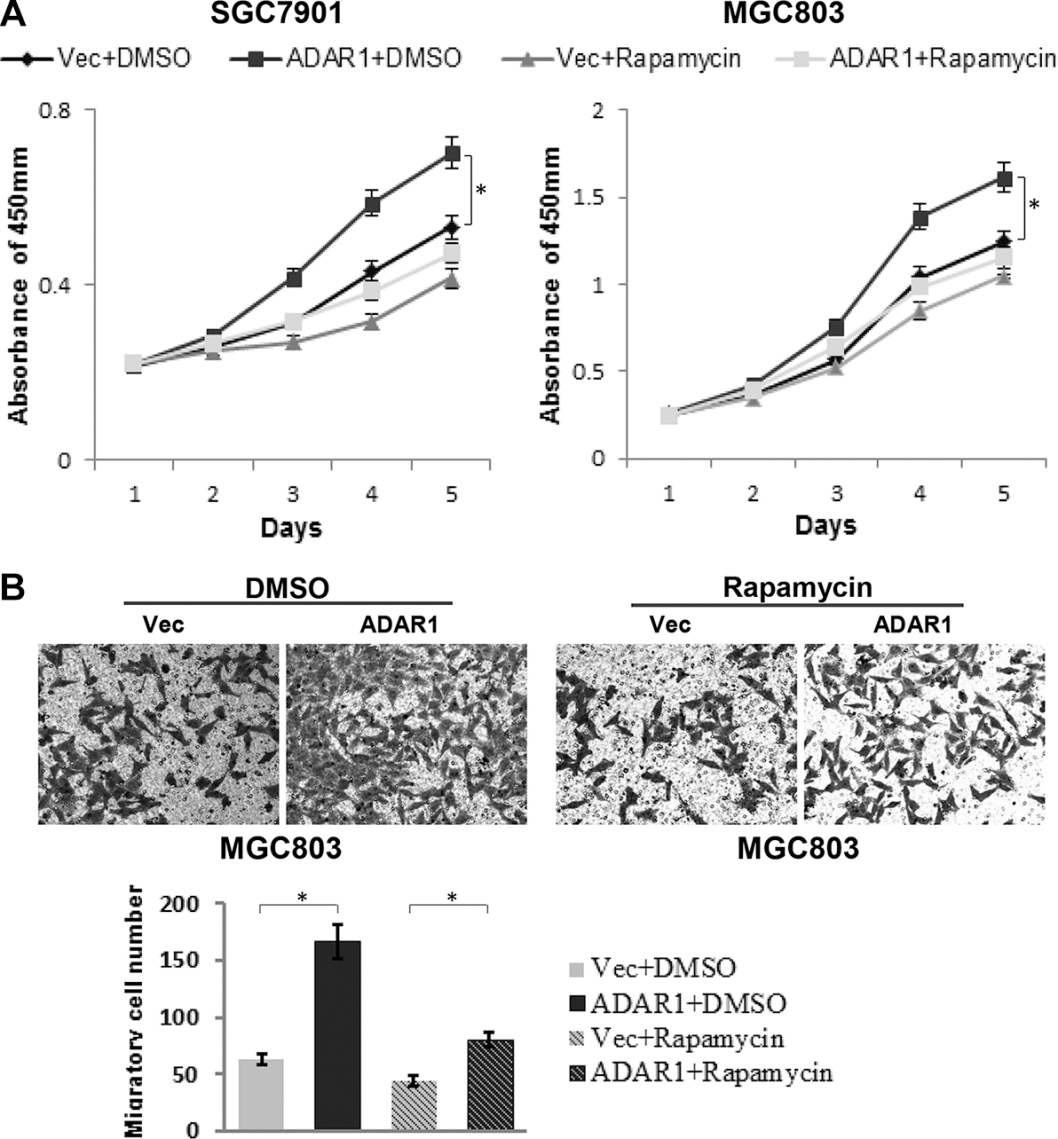
Rapamycin partially blocked the effects of ADAR1 overexpression on GC cell growth and migration (**A**) The effects of ADAR1 overexpression on SGC7901 and MGC803 cell proliferation with or without rapamycin treatment (10 μM), *p < 0.05. (**B**) Representative photographs of migratory cells on the transwell membrane (magnification, 200×). The below panel is the average MGC803 cell number of triplicate, *p < 0.05.

To address the functional relevance of ADAR1 and mTOR signaling, we used the mTOR kinase inhibitor, rapamycin, to treat GC cells and observe its effects on ADAR1-overexpressed cell proliferation and migration. The final data indicated that ADAR1 overexpression dramatically promoted cell proliferation and migration as expected, but these effects were significantly attenuated in rapamycin treated cells (Figure 7), demonstrating that rapamycin could block the effects of ADAR1 overexpression on GC cell growth and migration. These results suggested that mTOR signaling is important for ADAR1 mediated GC progression.

## DISCUSSION

ADAR1 functions as an RNA editing enzyme that catalyzes the deamination of A to I. The aberrant expression of ADAR1 leads to various diseases development due to the dysregulation of A to I RNA editing. Previous reports have shown that ADAR1 is involved in the regulation of tumorigenesis, however, little is known about its roles in gastric cancer. In the present study, we provided evidence that ADAR1 contributes to gastric cancer progression by promoting cell proliferation and migration. We found that ADAR1 is increased in GC samples and its high expression is closely associated with tumor stage, N classification and prognosis of GC patients, although there is no statistically significant relationship between ADAR1 expression and other clinicopathological variables such as age, gender or differentiation. Moreover, the results of flow cytometric analysis and EdU incorporation assay strongly support the roles of ADAR1 in the regulation of cell viability and proliferation. Consistent with our observations, a more recent report regarding ADAR-mediated RNA editing in GC progression revealed that ADAR1 plays an oncogenic and ADAR2 plays a tumor suppressive role in GC [30].

As a RNA editing enzyme, ADAR1 protein processes highly reproducible binding sites in whole genome. It is believed that ADAR1 binding sites should be enriched in Alu regions which contain majority of human A-to-I editing sites [31, 32]. Interestingly, a large fraction (15%) of binding sites is located in non-Alu regions [27], indicating that ADAR1 has diverse functional roles and diverse underlying mechanisms. In lung cancer, ADAR1 was reported to exert growth-enhancing activity through mediating the A-to-I editing levels of coding region in NEIL1 (a DNA repair enzyme) and non-coding region in pre-miR381 RNA transcripts [21]. ADAR1 generally works by forming two different types of complexes: homodimers and heterodimers [33]. The ADAR1 homodimers is in the well-known A-to-I editing mechanism while heterodimers, for example, Dicer/ADAR1 works as a regulator of microRNA processing and RNA-induced gene silencing [33]. The biological function of ADAR1 is supposed to be dependent on the balance of the two types of dimers. In addition, ADAR1 has two isoforms: p150 and p110. Our western blot results indicated that ADAR1 p150 protein is the dominant isoform in GC cell lines, in line with a previous study that p150 protein is essential for maintenance of proper cell growth in HeLa cells [34].

Up to date, there is no report about ADAR1 involved in signaling transduction pathway in cancer. In our study we found that ADAR1 affects mTOR signaling pathway activities in GC. Silencing of ADAR1 decreases phosphorylation level of mTOR, p70S6 kinase, and S6 ribosomal protein, whereas overexpression of ADAR1 increases phosphorylation level of these three proteins. The mTOR/p70S6K/S6 ribosomal protein axis is a key signaling pathway in control of protein translation, mRNA turnover, cell proliferation and mobility, actin cytoskeletal organization and autophagy [35, 36]. The mTOR protein, a highly conserved serine/threonine kinase, positively regulates the phosphorylation of p70S6 kinase, and S6 ribosomal protein is a well-known substrate of p70S6 kinase. Several studies have revealed an oncogenic potential for the mTOR/p70S6K pathway in tumorigenesis [37–39]. In our case, this signaling pathway may be downstream effector for ADAR1 in promoting GC progression. Thus, our data implicated a new mechanism for ADAR1 function in tumor. Most notably, the phosphorylation of Akt, an upstream activator of mTOR, was not changed dramatically whether in the PathScan^®^ intracellular signaling array or followed western blot analysis (data not shown). This implies the effect of ADAR1 on mTOR signaling is independently of Akt activity. With regard to the exact molecular mechanism of how ADAR1 regulates the phosphorylation level of these proteins, further study is deserved to do in the further.

In conclusion, our study suggests that ADAR1 functions as an oncogene in GC and may participate in mTOR/p70S6 kinase pathway for the first time. These findings provide new insights into the biologic and therapeutic relevance of ADAR1 in GC cell proliferation, survival and motility. ADAR1 is becoming a novel anti-tumor target for this disease therapy or a valuable biomarker for GC diagnosis and prognosis.

## MATERIALS AND METHODS

### Human gastric cancer specimens

Human gastric cancer and adjacent gastric mucosa specimens were collected from 38 patients at Shanghai East hospital. Written informed consent was obtained from all participants and study protocol was approved by the ethics committee of Shanghai East Hospital, Tongji Univeristy School of Medicine. After resection, all the samples were snap-frozen in liquid nitrogen and stored at −80°C prior to RNA extraction. Human gastric cancer tissue microarray (ST2091) containing 208 patients’ sample was purchased from Xian Alenabio Company. Among these patients, 64 were women and 144 were men, with an age range between 17 and 84 years old. ADAR1 expression was assessed by immunohistochemical staining using an anti-ADAR1 antibody (ab126745, Abcam) at a dilution of 1:500. The staining results were classified according to the percentage of positive cells and staining intensity: negative (−), slight positive (+), moderate positive (++), or strong positive (+++). To prevent bias from knowledge of clinical data, scoring of staining intensity was conducted in a blinded manner.

### Cell lines and culture conditions

Five human gastric cancer cell lines AGS, HGC27, SGC7901, BGC823 and MGC803 were obtained from Shanghai Cell Bank of Chinese Academy of Sciences. Cells were cultured at 37°C with 5% CO_2_ and maintained in Modified Eagle's medium (MEM, Corning, US) supplemented with 10% fetal bovine serum and 100 μg/mL penicillin/streptomycin. Rapamycin (S1039) was purchased from Selleck Chemicals, US.

### RNA extraction and quantitative real-time PCR

Total RNA was isolated from GC samples and cells with RNAiso Plus reagent (TaKaRa, Japan) according to the manufacturer's protocol. RNA was eluted with RNase-free water and stored at −80°C. Reverse transcription was performed with the PrimeScriptTM RT Reagent Kit with gDNA Eraser (TaKaRa, Japan) and SYBR green reagent (TaKaRa, Japan) on an ABI 7500 Real Time System. The 2^-ΔΔCt^ method was used for quantification, and b-actin was used as an endogenous control. Each experiment was conducted for at least three times. The following primer pairs were used to amplify and measure the amount of ADAR1 and β-actin: ADAR1-qF:5′-AAGTCCTGCAGCGACCGTGC-3′, ADAR1-qR:5′-TCTCCCCGAGCCGAATGCCA-3′ and β-actin-qF: 5-CCTGGCACCCAGCACAATG-3, β-actin-R: 5-GGGCCGGACTCGTCATACT-3.

### RNA interference, plasmid construction and transfection

Transient knockdown of ADAR1 was performed with two different siRNAs against ADAR1, which were designed and synthesized by GenePharma, Shanghai, China. The sequences were designed as follow: siADAR1-1 (sense 5-CGCA GAGUUCCUCACCUGUAdTdT-3), siADAR1-2 (sense 5-GCCAAGGUUUCCAGUACUAdTdT-3) and a non-specific control siNC (sense 5-UUCUCCGAACGUG UCACGUdTdT-3). To overexpress ADAR1, pEnter-ADAR1 (GeneBank Accession Number: NM_001111) was purchased from Vigene Biosciences, Shandong, China. Cell transfection with plasmids or siRNAs was conducted using Lipofectamine 3000 (Invitrogen, USA) in accordance with the manufacturer's instructions. AGS, HGC27, SGC7901 and BGC823 cells were infected with the lentivirus knocking down ADAR1 or control (LV-shADAR1, LV-shNC). Each lentivirus was packaged and purchased from GenePharma, Shanghai using above corresponding sequences (siADAR1-1, siNC). Stably transfected cell lines were isolated by puromycin selection.

### Cell migration

Cell migration was conducted using a modified 24-well Boyden chamber with a membrane. 24 h after transfection with plasmids or stably infected with LV-shADAR1, the cells were harvested and resuspended in MEM free FBS. A 400 μL of cells were loaded in the upper wells, and a medium containing 5% FBS was placed in the lower wells as a chemoattractant stimulus. Migrated cells on the bottom surface of the filter were fixed, stained with 0.5% crystal violet, and counted in five random fields under a microscope and the average number of five fields was calculated. All assays were performed in triplicate and repeated three times.

### Western blot analysis

The gastric cancer tissues and cells were cracked in lysis buffer for 30 minutes on ice and then centrifuged at 12000 × rpm for 10 min to collect the supernatant. The supernatant was diluted in 5 × SDS loading buffer and boiled. An equivalent amount of proteins from each sample were electrophoresed by 10% sodium dodecyl sulfatepolyacrylamide gel electrophoresis (SDS-PAGE) and then transferred on to a polyvinylidene difluoride membrane (EMD Millipore, USA). After that, the membranes were blocked in non-fat milk for about 1 h at room temperature and then incubated 2 h at room temperature or overnight at 4°C with the primary antibodies. After washing with Phosphate Buffered Saline (PBS) containing 0.05% Tween 20 three times, each for 5 min, the membranes were then incubated with the secondary antibody for another 1 h at room temperature. Detection of proteins was achieved by using the Odyssey Infared Imaging System (Li-COR, USA) according to the manufacturer's instructions. The antibodies used in this study included: anti-ADAR1 (1:1000, abcam, #126745), β-actin (1:500, Santa Cruz Biotechnology, #81171), GAPDH (1:20000, Proteintech, 10494-1-AP), p70S6, Kinase (1:1000, Proteintech, #14485).mTOR (7C10), Phospho-mTOR (Ser2448) (1:1000, Cell Signaling Technology, #9864), phospho-p70S6 Kinase(Thr389) (1:1000, Cell Signaling Technology, #9234),Phospho-S6 Ribosomal Protein (Ser235/236) (1:1000, Cell Signaling Technology, #4858), S6 Ribosomal Protein (54D2) (1:1000, Cell Signaling Technology, #2317).

### Cell proliferation

Twenty-four hours after transfection, cells (3,000 cells/well) were seeded in 96-well plates in triplicate and maintained in MEM containing 10% FBS for 5 days. According to the manufacturer's instructions, 10 μL of Cell Counting Kit-8 reagent (CCK8, Dojindo, Kumamoto, Japan) was added to each well for 1 h incubation at 37°C. The absorbance was read at the wavelength of 450 nm in an automated plate reader. All experiments were independently repeated at least three times.

### Colony formation assays

Stably infected cells (AGS-LV-shNC, AGS-LV-shADAR1; HGC27LV-shNC, HGC27-LV-shADAR1; BGC823-LV-shNC, BGC823-LV-shADAR1) were cultivated in 6-well culture plates at a density of 2000 cells/well. After two weeks, the cell colonies (> 50 cells/colony) were photographed and counted by staining with 0.5% crystal violet.

### Wound healing assay

The stably expressed shADAR1 or control cells were seeded into 6-well plates to achieve 90% confluence and incubated at 37°C with 5% CO_2_. Cell monolayer was scratched with a plastic tip, cell debris were removed using PBS, and 0.5% FBS-containing MEM was added. The wound gap was observed (per 24 h) and photographed by the light microscope (Nikon, Japan).

### EdU labeling and immunofluorescence

The EdU incorporation assay was performed as described previously [40]. Briefly, GC cells were seeded in 24-well culture plates. After 24 h, cells were incubated with 50 mM 5-ethynyl-2′-deoxyuridine (EdU, RIBOBIO, China) for 2 h and stained with Apollo^®^567 according to the manufacturer's instruction. The stained cells were observed with microscope and counted.

### Flow cytometry analysis

For cell apoptotic analysis, cells was performed using the Annexin V-FITC/PI apoptosis detection kit (KGA-107, KeyGEN, Nanjing, China) according to the manufacturer's instructions. Data was analyzed by flow cytometer (BD Bioscience).

### Tumorigenicity assay in nude mice

For xenograft model of gastric cancer, four to five-week-old BALB/c nude mice were purchased from the Sippr-BK laboratory animal corporation, Shanghai, China, two group of stably infected cells (BGC823-LV-shNC, BGC823-LV-shADAR1) were subcutaneously injected into the dorsal flank of nude mice (*n* = 5 per group), respectively. The weight of the resulting tumors was measured after 2 weeks. For *in vivo* tumor metastasis, mice tail vein–injected with 1.5 × 10^6^ cells (7901-LV-shNC, SGC7901-LV-shADAR; *n* = 6 per group, BALB/c, male, 4–5 weeks of age). After injection (40 days), mice were sacrificed and the visible tumor nodules on the lung surface were calculated. In addition, these lung tissues were fixed in 4% paraformaldehyde, and paraffin sections were made and stained with hematoxylin and eosin (H&E). All animal handling and experimental procedures were approved by the Ethics Committee of Shanghai East Hospital.

### Path scan intracellular signaling array

To detect the activation of intracellular signaling, the PathScan intracellular signaling array was used. Intracellular signaling was detected using a primary PathScan^®^ Intracellular Signaling Array Kit #7744(Cell Signaling Technology) following the manufacturer's instructions. Data was quantified by Image Studio Ver3.1 and results were further detected by western blot.

### Statistical analysis

Quantitative data were presented as mean ± SD. The χ2 test was used to determine the significance of the difference among the covariates. The significance of the *in vitro* data was determined using the Student *t-test* (two-tailed). Only a *P value* of less than 0.05 was considered significant. “*” indicates *P* < 0.05; “**” indicates *P* < 0.01.
